# Suan-Zao-Ren Decoction ameliorates synaptic plasticity through inhibition of the Aβ deposition and JAK2/STAT3 signaling pathway in AD model of APP/PS1 transgenic mice

**DOI:** 10.1186/s13020-021-00425-2

**Published:** 2021-01-21

**Authors:** Qing-Hua Long, Yong-Gui Wu, Li-Ling He, Li Ding, Ai-Hua Tan, He-Yuan Shi, Ping Wang

**Affiliations:** 1 School of Basic Medicine, Hubei University of Chinese Medicine, Wuhan, 430065 Hubei China; 2 School of Pharmacy, Hubei University of Chinese Medicine, Wuhan, 430065 Hubei China

**Keywords:** Suan-Zao-Ren Decoction, Alzheimer’s disease, Synaptic plasticity, Neuroinflammation, APP/PS1 transgenic mice, JAK2/STAT3 pathway

## Abstract

**Background:**

Suan-Zao-Ren Decoction (SZRD) has been widely used to treat neurological illnesses, including dementia, insomnia and depression. However, the mechanisms underlying SZRD’s improvement in cognitive function remain unclear. In this study, we examined SZRD’s effect on APP/PS1 transgenic mice and mechanisms associated with SZRD’s action in alleviating neuroinflammation and improving synaptic plasticity.

**Methods:**

The APP/PS1 mice were treated with different dosages of SZRD (12.96 and 25.92 g/kg/day, in L-SZRD and H-SZRD groups, respectively) for 4 weeks. Morris water maze was conducted to determine changes in behaviors of the mice after the treatment. Meanwhile, in the samples of the hippocampus, Nissl staining and Golgi-Cox staining were used to detect synaptic plasticity. ELISA was applied to assess the expression levels of Aβ_1−40_ and Aβ_1−42_ in the hippocampus of mice. Western blot (WB) was employed to test the protein expression level of Aβ_1−42_, APP, ADAM10, BACE1, PS1, IDE, IBA1, GFAP, PSD95 and SYN, as well as the expressions of JAK2, STAT3 and their phosphorylation patterns to detect the involvement of JAK2/STAT3 pathway. Besides, we examined the serum and hippocampal contents of IL-1β, IL-6 and TNF-α through ELISA.

**Results:**

Compared to the APP/PS1 mice without any treatment, SZRD, especially the L-SZRD, significantly ameliorated cognitive impairment of the APP/PS1 mice with decreases in the loss of neurons and Aβ plaque deposition as well as improvement of synaptic plasticity in the hippocampus (*P* < 0.05 or 0.01). Also, SZRD, in particular, the L-SZRD markedly inhibited the serum and hippocampal concentrations of IL-6, IL-1β and TNF-α, while reducing the expression of p-JAK2-Tyr1007 and p-STAT3-Tyr705 in the hippocampus of the APP/PS1 mice (*P* < 0.05 or 0.01).

**Conclusions:**

The SZRD, especially the L-SZRD, may improve the cognitive impairment and ameliorate the neural degeneration in APP/PS1 transgenic mice through inhibiting Aβ accumulation and neuroinflammation via the JAK2/STAT3 pathway.

## Introduction

Alzheimer’s disease (AD) is a chronic degenerative illness in the brain and is one of the common kinds of dementia [1, 2]. Based on the AD’s World Report 2018, the patients with AD worldwide exceeded 47 million in 2018 [3]. In other words, one patient is diagnosed with dementia every 3 s [4]. With the growth of the aging population, the AD prevalence in the world is increasing rapidly. It is estimated that AD patients in the world will be more than 150 million by 2050 [2]. Currently, the drugs used for AD treatment are composed of Donepezil, rivastigmine, galanthamine and memantine [5, 6]. But their therapeutic effects are limited. These drugs can only alleviate AD-associated cognitive impairment, but they cannot fundamentally control or reverse the AD pathogenesis [7]. Thus, AD has become a public health problem, and its prevention and treatment are becoming more and more critical.

One typical pathological character of AD is the formation of senile plaques in the brain, which are induced by amyloid-β (Aβ) deposition and neurofibrillary tangles generated by exaggerated phosphorylation of tau protein within the neuron [8–10]. In addition, neuron loss and synaptic dysfunction mainly caused by senile plaques and neurofibrillary tangles are another crucial pathological feature of AD [11]. The causes of neuron loss and synaptic dysfunction of AD are complex, involving multiple factors such as neurotoxic Aβ, neurofibrillary tangles and neuroinflammation [12]. The inflammation is a double-edged sword for the pathological process of AD [13, 14]. On the one hand, inflammation can clear Aβ deposition, and On the other hand, inflammation can also damage neurons and synapses and thereby accelerates the AD’s pathological processes [15]. Although the AD’s pathogenesis is not elucidated clearly, the inflammation likely is involved in the AD’s pathological process [16]. Traditionally, microglia are activated in neuroinflammatory conditions to release pro-inflammatory factors to accelerate AD’s progression and pathology [17]. An epidemiological study has shown that the administration of non-steroidal anti-inflammatory drugs (NSAIDs) can lower AD’s prevalence [18]. Several other studies have confirmed that the pro-inflammatory factors, especially the TNF-α, IL-1β and IL-6, are significantly increased in the AD experimental models [19–21]. Aβ deposition can cause neuronal loss and synaptic damage [22]. Also, neuroinflammation induced by Aβ deposition aggravates the loss of neurons and the dysfunction of synapses [23]. Therefore, inhibition of Aβ deposition and neuroinflammation may be beneficial to AD’s management.

Janus kinase2 (JAK2)/Signal transducer and activator of transcription3 (STAT3) are involved in an important signaling pathway that regulates inflammation in the brain [23]. Recently, some studies have shown that abnormal activation of the signaling pathway associated with JAK2/STAT3 happens in AD patients [24, 25]. Therefore, the modulation of the signaling pathway related to JAK2/STAT3 in the brain may effectively inhibit the AD’s pathological process.

Suan-Zao-Ren Decoction (SZRD), a well-known classic Chinese formula, was created by Zhang Zhongjing in Han Dynasty. SZRD consists of five Chinese herbal medications, including Ziziphi Spinosae Semem (Suanzaoren), Poria (Fuling), Chuanxiong Rhizoma (Chuanxiong), Anemarrhenae Rhizoma (Zhimu) and Glycyrrhizae Radix Et Praeparata Cum Melle (Zhigancao).

Since the Han Dynasty, SZRD has been widely used to treat neurological disorders, such as dementia, insomnia and depression [26–28]. It effectively improves the learning and memory of AD patients. These are studies showing that SZRD has a potential benefit for AD treatment. In particular, our previous research has demonstrated that SZRD improves cognitive dysfunction through inhibiting neuroinflammation in APP/PS1 transgenic mice [29, 30]. However, pathological mechanisms underlying SZRD’s anti-neuroinflammation remain indefinite. In this study, we examined SZRD’s effect on APP/PS1 transgenic mice and investigated mechanisms by which SZRD exerts anti-neuroinflammation and improves synaptic plasticity.

## Methods and materials

### Animals

Forty male APP/PS1 transgenic mice with Specific pathogen-free (SPF), weighing about 30 g, were purchased from Beijing HFK Bioscience Co., Ltd. (Certification number SCXK 2014-0004). Six-month-old male C57BL/6J mice were bought from Beijing Vital River Laboratory Animal Technology Co., Ltd. (Certification number SCXK 2016-0006). These mice were housed in the Laboratory Animal Center at the Hubei University of Chinese Medicine. They lived with a 12-h light/dark cycle under 23–25 ℃, meeting clean experimental animal feeding standards. All animals freely accessed food and water. Experimental protocols were permitted by the Chinese Association of Accreditation of Laboratory Animal Care and performed following the ethical guidelines.

### Drugs

SZRD contains five Chinese herbal medications, namely, Ziziphi Spinosae Semem (Suanzaoren, 30 g), Poria (Fuling, 6 g), Chuanxiong Rhizoma (Chuanxiong, 6 g), Anemarrhenae Rhizoma (Zhimu, 9 g) and Glycyrrhizae Radix Et Praeparata Cum Melle (Zhigancao, 3 g), which were obtained from Jiuzhou Tong Pharmaceutical Group Co., Ltd. (Hubei, China). Donepezil hydrochloride (Aricept, 5 mg/tablet, Batch number: 1706065) was produced by Eisai Pharmaceutical Co., Ltd. (Jiangsu, China).

### Preparation of SZRD extract

All herbal ingredients were confirmed by the Department of Chinese Medicine pharmacy at the Hubei University of Chinese Medicine. Referred to Gao’s report, the stock solution of SZRD was prepared [6]. Briefly, the raw herbs were immersed in water 14 times of the volume of traditional Chinese medicine for 30 min, then boiled and filtered, and finally made into a stock solution.

### Reagents and antibodies

Nissl staining solution was purchased from Servicebio (Wuhan, China; G1036). GolgiStain™ Kit was purchased from FD Neurotechnologies, Inc., (Columbia, USA; #PK401). ELISA Kits were obtained from Cusabio Biotech Co., Ltd. (Wuhan, China; CSB-E08300m, CSB-E10787m, CSB-E08054m, CSB-E08054m and CSB-E04639m, respectively). They were used to examining Amyloid beta peptide 1–40 (Aβ_1−40_), Amyloid beta peptide 1–42 (Aβ_1−42_), Interleukin 6 (IL-6), Interleukin 1β (IL-1β), and tumor necrosis factor-α (TNF-α) in the mouse. The antibodies and their epitopes on the protein molecules are displayed in Table 1, which were used for Western blot in this study.

**Table 1:** A list of antibodies and their epitopes on the molecule of protein used in this study

Antibody	Specificity	Type	Dilution	Source	Catalog No.
Aβ_1−42_	Rabbit	Mono-	1:1000 for WB	ABcam	ab201060
APP	Rabbit	Poly-	1:1000 for WB	ABclonal	A16265
ADAM10	Rabbit	Poly-	1:1000 for WB	ABclonal	A10438
BACE1	Rabbit	Poly-	1:1000 for WB	ABclonal	A5266
PS1	Rabbit	Mono-	1:5000 for WB	ABcam	ab76083
IDE	Rabbit	Poly-	1:1000 for WB	ABclonal	A1630
PSD95	Rabbit	Mono-	1:1000 for WB	ABclonal	A10841
SYN	Rabbit	Mono-	1:1000 for WB	ABclonal	ab32127
IBA1	Rabbit	Poly-	1:1000 for WB	ABcam	A12391
GFAP	Rabbit	Poly-	1:1000 for WB	ABclonal	A0237
JAK2	Rabbit	Mono-	1:1000 for WB	ABclonal	A19629
p-JAK2-Tyr1007	Rabbit	Poly-	1:1000 for WB	ABclonal	AP0373
STAT3	Rabbit	Poly-	1:1000 for WB	ABclonal	A1192
p-STST3-Tyr705	Rabbit	Poly-	1:1000 for WB	ABclonal	AP0070
β-actin	Rabbit	Poly-	1:1000 for WB	ABcam	ab5694

Mono-, monoclonal; Poly-, polyclonal; WB, Western blot

### Experimental groups

Based on the randomization table, the 40 APP/PS1 transgenic mice were allocated randomly into four experimental groups, and each group included ten mice. These groups include: model group, donepezil group, L-SZRD (low-dose SZRD) group and H-SZRD (high-dose SZRD) group. The donepezil group was given the donepezil hydrochloride (2 mg/kg/day) on the basis of previous report [31]. According to our previous reports and clinical equivalent doses, in which L-SZRD group (12.96 g/kg/day) was given clinically equivalent dose, and H-SZRD group (25.92 g/kg/day) was administered twice clinically equivalent dose [29, 30]. Besides, ten of C57BL/6J mice were assigned to a control group. Intragastric administration of the drug in each group was performed in a volume of 0.2 ml/10 g/day, once a day for 4 weeks. The same volume of normal saline was taken in the mice in the control and model group.

### Morris water maze

Morris water maze (MWM) test is a major behavioral testing method for measuring cognitive impairment of rodents, and it has been widely used to evaluate the cognitive function of neurodegenerative diseases, including AD and Parkinson’s disease (PD) [32]. Thus, in this study, we used the MWM to examine mice’s cognitive function, especially the spatial learning and memory, after 4-week treatments. The MWM contains a pool, a hidden platform, and a video/computer system (version ZH-Morris; Anhui Zhenghua Biological Instrument Co., Ltd.; Anhui, China). The MWM test includes the navigation and spatial probe tests, which were modified as described previously [8]. Briefly, during the navigation test, the mice were trained to escape onto the platform within 60 s in 5 consecutive days. If the animal could not find the platform in the permitted 60 s, it was directed to the platform and stayed on the platform for at least 15 s. On the 7th day, we removed the platform from the pool and carried out the spatial probe test. The video/computer system automatically taped the mouse’s movement, the number of times as the mouse crossed the stage within 60 s was documented.

### Preparation of the serum and hippocampus samples

Following the MWM test, four animals in each group were sacrificed by CO_2_ asphyxiation followed by cervical dislocation, and the brain tissues were split into two parts. One portion of the brain was fixed by 4 % paraformaldehyde for Nissl Staining and another part was treated with the Golgi-Cox staining solution for the Golgi-Cox staining. The blood of the remaining six mice in each group was collected by removing eyeball. Afterward, the blood was centrifuged (3000 rpm) for 10 min at 4 ℃. The serum was gathered and stored at − 80 ℃. The hippocampus tissue was separated on a cold plate and put in liquid nitrogen. It was then kept at − 80 °C until use.

### Nissl staining

After the mouse’s brain was fixed with 4 % paraformaldehyde for 24 h, it then was implanted into paraffin. The paraffin-embedded brain sample was serially sliced into sections (6 µm) with a microtome (CM1860S, Leica, Germany). The brain section was dewaxed with xylene and gradient ethanol. It then was rinsed with PBS solution twice, 5 min for each wash. After dustproof atmospheric drying for 24 h, Nissl staining solution was uniformly added to the brain slices, and 95 % alcohol was then dropped on the slices to differentiate for 8–10 min. Following rinsing with PBS solution twice (5 min for each time), the brain slices were dehydrated with anhydrous alcohol for 15 min, permeabilized with xylene for 15 min, and then sealed with neutral gum. Finally, Neurons and Nissl bodies in the hippocampus were observed under a biological microscope, and the images were collected for analysis.

### Golgi-Cox staining

According to the methods described by Jiang and the instructions for using a Rapid Golgi Stain Kit (FD Neuro-Technologies, Inc., Ellicott City, MD, USA), Golgi-Cox staining was conducted with the staining Kit [33]. Briefly, at room temperature, brain tissues were submerged in a mixture of solution A and solution B for 2 weeks. They were then put into solution C for 2 to 7 days. Afterward, the brains were sliced into sections (100 µm) using a microtome (CM1860S, Leica, Germany) and pasted on the glass slide. After washing with double-distilled water for 4 min × 2, the slides were immersed in a blend of solution C, solution D and double-purified water.

### Enzyme‐linked immunosorbent assay

We used enzyme-linked immunosorbent assay (ELISA) to measure the contents of IL-1β, IL-6 and TNF-α in the serum and hippocampus. After thawing the serum samples at room temperature, the serum IL-1β, IL-6 and TNF-α were measured through the specific ELISA kit for each of them. Each sample was measured in 450 nm wavelength with Microplate Reader (Spectra MAX M5, Molecular Devices, USA). In addition, we used ELISA to measure the expression levels of Aβ_1−40_ and Aβ_1−42_ in the hippocampus of mice. At first, we used the BCA protein kit to quantify the protein in the hippocampus of each sample, we then detect the levels of Aβ_1−40_ and Aβ_1−42_ in the hippocampal tissue, as described in the ELISA kit instructions.

### Western blot

We used Western blot to detect expressions of Aβ_1−42_, APP, ADAM10, BACE1, PS1, IDE, IBA1, GFAP, PSD95, SYN, JAK2, p-JAK2, STAT3, p-STAT3 and β-actin in the mouse’s hippocampus. In each group, six samples were homogenized using RIPA lysis buffer. The homogenates were subjected to centrifugation (12,000 rpm) at 4 ℃ for 20 min to obtain proteins. The BCA kit was used to detect protein concentration. Afterward, the protein was isolated using 10 % SDS-PAGE, and it then was transferred to a Polyvinylidenefluoride (PVDF) membrane (EMD Millipore, Billerica, MA, USA). After reacting with the primary antibody overnight at 4 ℃, the membrane was immersed in a horseradish peroxidase-conjugated secondary antibody for 2 h at room temperature. Subsequently, the greyscale images were obtained and analyzed with Image J software (v1.80; NIH, Bethesda, MD, USA).

### Statistical analysis

Experimental data were analyzed by using SPSS 19.0 (IBM Corporation, Armonk, NY, USA), and the data were presented as mean ± standard error (SEM). One-way ANOVA was used to make comparisons among multiple groups. If the variance was homogeneous, the LSD tests were used. If not, the Dunnett T3testswere conducted. Values were considered to be significantly different as *P* < 0.05.

## Results

### The action of SZRD in ameliorating cognitive dysfunction in APP/PS1 transgenic mice

**Fig. 1:**
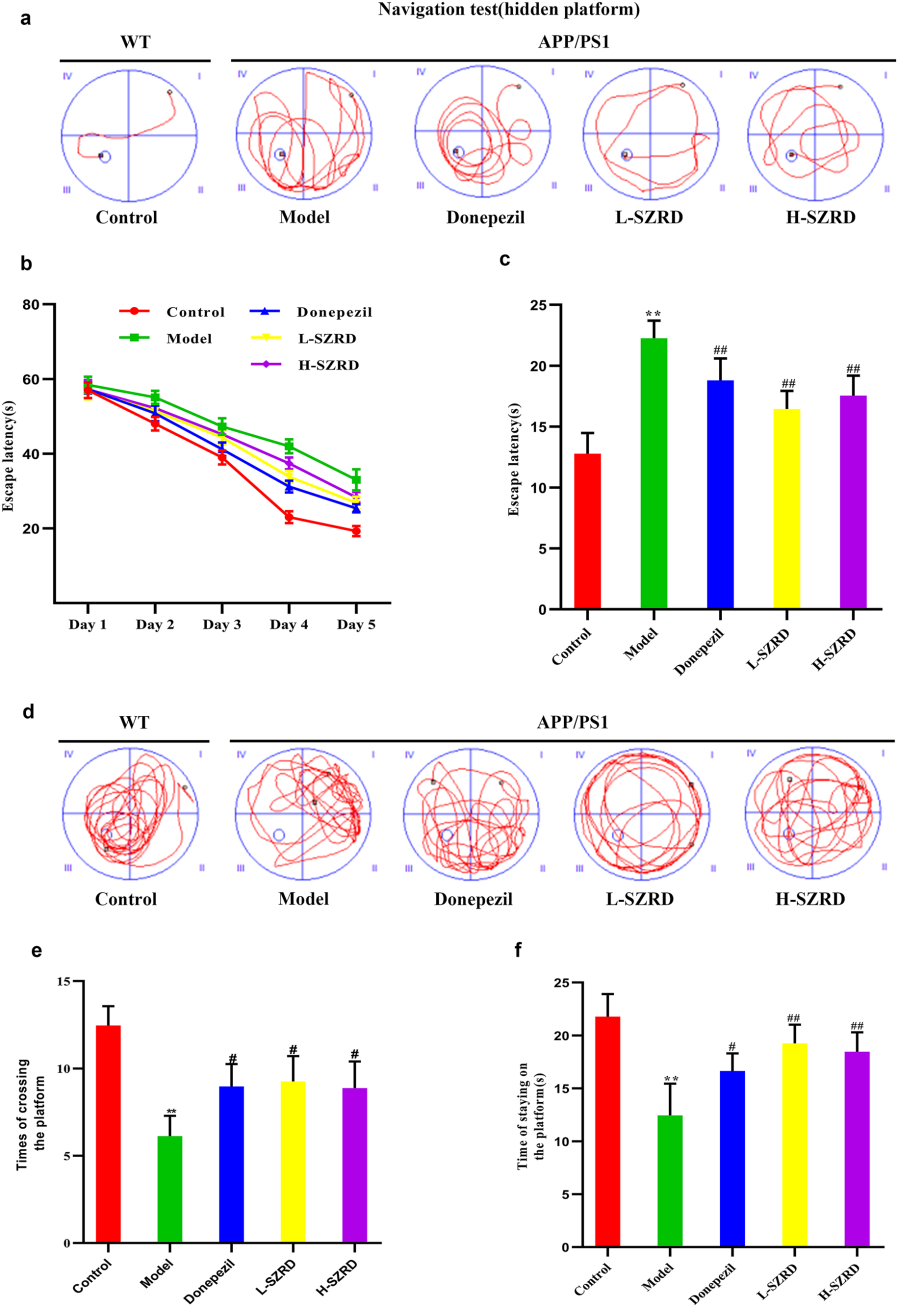
The effect of SZRD on cognitive dysfunction in APP/PS1 transgenic mice. **a** Representative tracings of the mice’s movements in the presence of the hidden platform, which were generated from the navigation test. **b** Mice’s escape latencies in each day during the 5 consecutive days. **c** Bar graphs showing the escape latencies of mice in each group. **d** Representative tracings of the mice’s movements in the absence of the hidden platform, which were obtained from the probe test. **e** The time of the mice staying on the stage within 60 s. **f** The searching time that the mice stayed in the target quadrant. The data are presented as mean ± SEM (n = 10 in each group). **P < 0.01 vs. Control group; ^#^*P* < 0.05 and ^##^*P* < 0.01 vs. Model group

In this study, we used the navigation test and the probe test was to evaluate the spatial learning ability and the spatial memory ability of mice, respectively. In the navigation test, in comparison with the model group, the escape latency was markedly shorter in the Donepezil group and SZRD-treated groups, particularly in the L-SZRD group (*P* < 0.01; Fig. 1a, c). No considerable alteration in the escape latency was noticed during the training from the 1st to 3rd days among all groups. However, starting from the 4th day, mice in the SZRD-treated groups had much shorter escape latency than animals in the model group (*P* < 0.01; Fig. 1b, c). In the probe test, compared to the control group, the times of crossing the platform and the time of staying on the platform were decreased in the model group (*P* < 0.05 or 0.01; Fig. 1d–f). However, the Donepezil and SZRD significantly increased the times of crossing the platform and the time of staying on the platform (*P* < 0.05 or 0.01; Fig. 1d–f). Although the H-SZRD group had longer times of crossing the platform and staying on the platform, there was no significant difference between these two groups. The above findings indicated that the SZRD, especially the L-SZRD can ameliorate learning and memory deficits in APP/PS1 transgenic mice.

### SZRD increases the hippocampal neurons in CA3 and DG of APP/PS1 transgenic mice

**Fig. 2:**
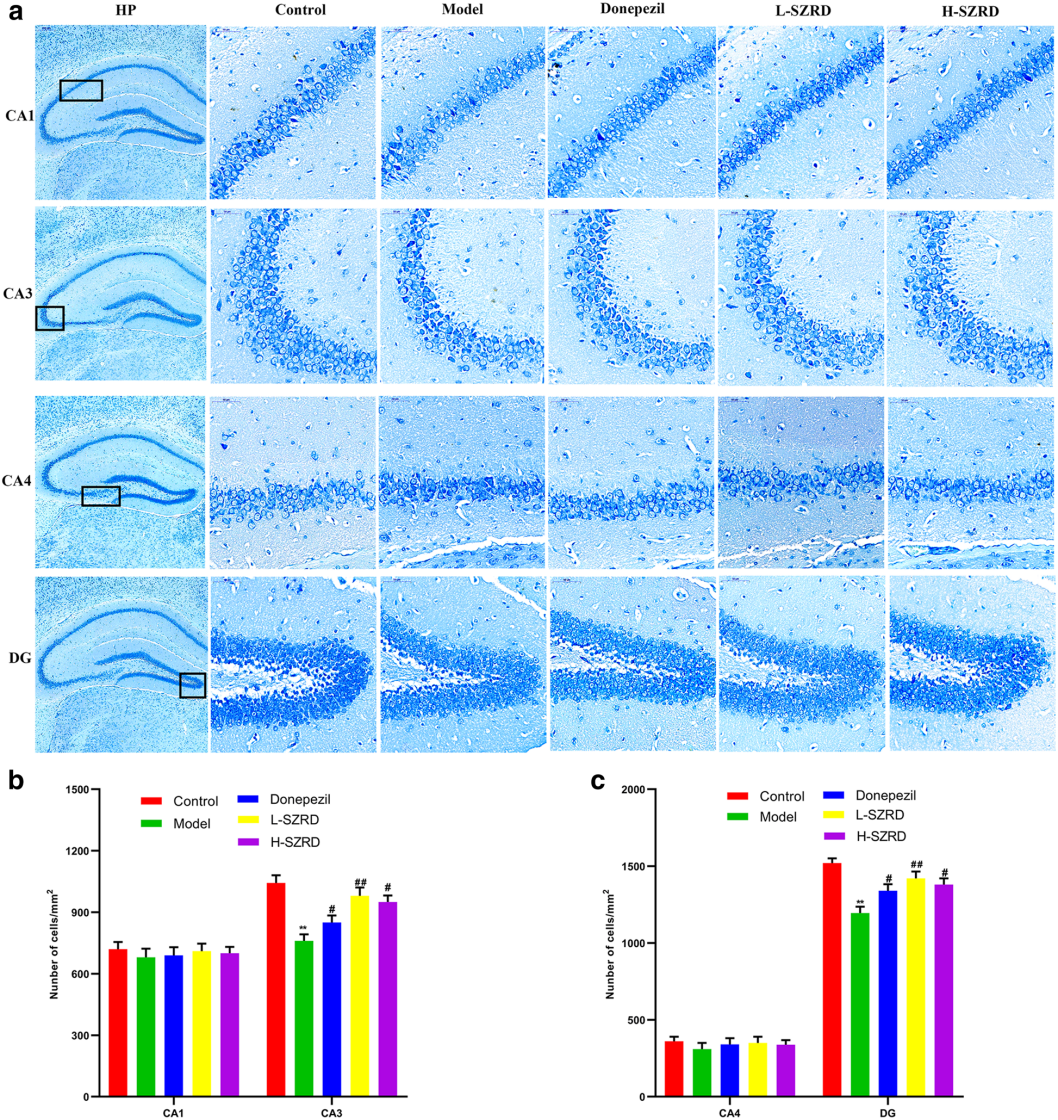
Changes in neuronal loss in hippocampal CA3 and DG of APP/PS1 transgenic mice treated with and without SZRD. **a** Microscopic images show neuronal labels by Nissl staining in the hippocampus after treatment with and without SZRD for 4 weeks (× 400 magnifications; scale bar represents 50 µm). **b** The neuronal number in the regions of CA1 and CA3. **c** The neuronal number in the regions of CA4 and DG. The data are presented as mean ± SEM (n = 4 in each group). **P < 0.01 vs. Control group; ^#^*P* < 0.05 and ^##^*P* < 0.01 vs. Model group

AD’s pathological feature is the loss of in hippocampal neurons [34]. Hippocampal neurons are the biological basis of hippocampal-dependent learning and memory. To investigate whether SZRD can improve the cognitive dysfunction of AD by inhibiting the loss of hippocampal neurons, we used Nissl staining to measure neurons in the hippocampus of mice. As shown in Fig. 2, compared to controls, the hippocampal neurons in the CA3 and DG were lost significantly in APP/PS1 transgenic mice (*P* < 0.01; Fig. 2a–c). However, in comparison with these transgenic mice in the model group, more neurons in CA3 and DG subsets were found in the Donepezil group and two SZRD-treated groups (*P* < 0.05 or 0.01; Fig. 2a–c). L-SZRD or H-SZRD had similar effects, and there were no significant differences between the L-SZRD and H-SZRD group in increasing hippocampal neurons in the CA3 and DG subsets. Besides, we did not observe substantial neuronal loss in the CA1 and CA4 subsets of all groups (Fig. 2a–c). All the results suggested SZRD may alleviate neuronal loss in CA3 and DG subsets of APP/PS1 transgenic mice.

### Effect of SZRD on the synaptic plasticity of APP/PS1 transgenic mice

**Fig. 3:**
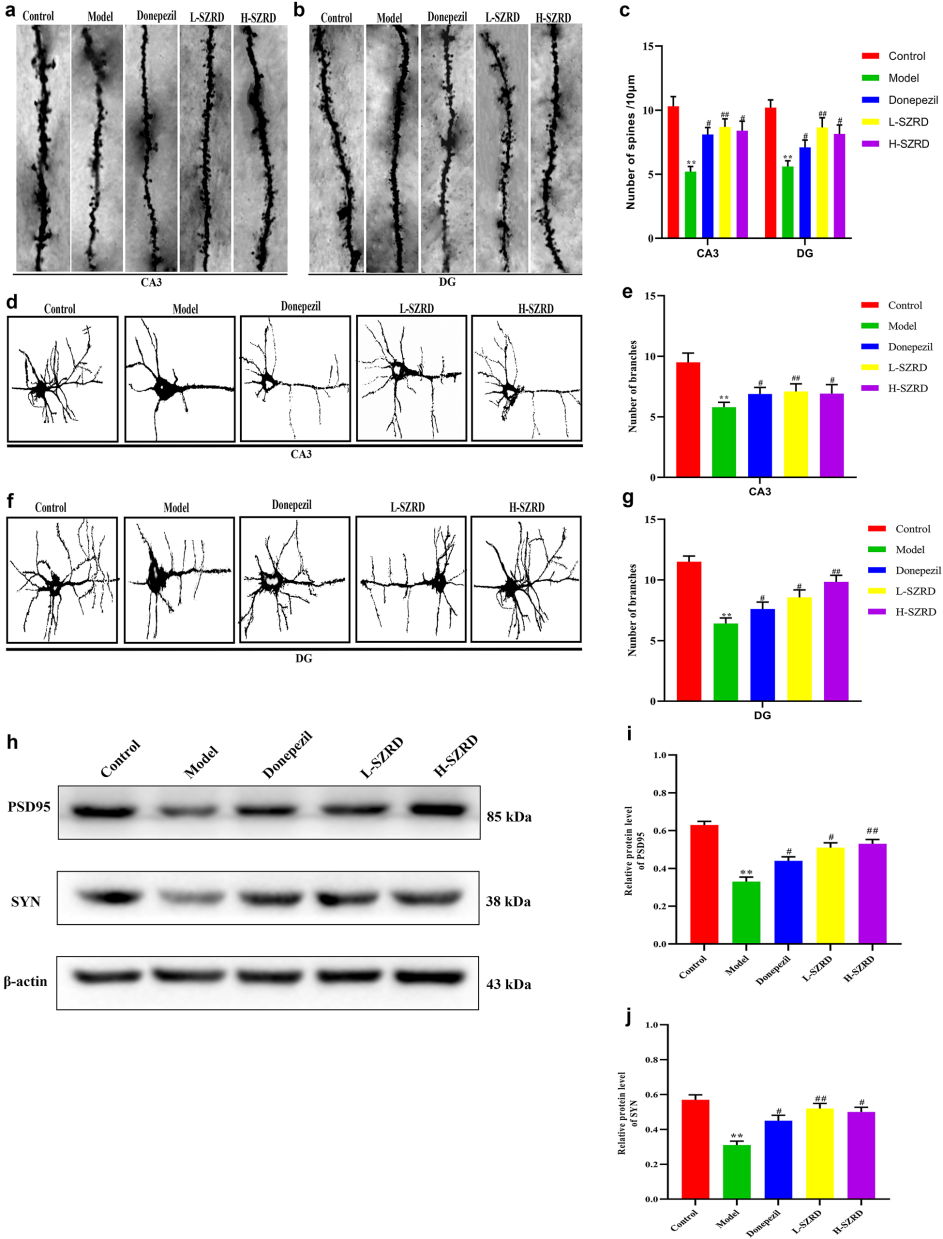
Effect of SZRD on the synaptic plasticity of APP/PS1 transgenic mice. **a** Representative Golgi-Cox staining in the hippocampus of CA3 after treatment with and without SRZD for 4 weeks (magnification, × 400; scale bar represents 20 µm). **b** Representative Golgi-Cox staining in the hippocampus of DG after treatment with and without SRZD for 4 weeks (magnification, × 400; scale bar represents 20 µm). **c** The number of spines in the areas of CA3 and DG. **d** Representative Golgi-Cox staining in the hippocampus of CA3 after treatment with and without SRZD for 4 weeks (magnification, × 400; scale bar represents 20 µm). **e** The number of spines in the areas of CA3. **f** Representative Golgi-Cox staining in the hippocampus of DG after treatment with and without SRZD for 4 weeks (magnification, × 400; scale bar represents 20 µm). **g** The number of spines in the areas of DG. **h** Examples of original Western blotting bands showing expressions of PSD95 and SYN in the hippocampus. **i** Relative protein level of PSD95. **j** Relative protein level of SYN. The data are presented as mean ± SEM (n = 4 in each group). **P < 0.01 vs. Control group; ^#^*P* < 0.05 and ^##^*P* < 0.01 vs. Model group

The Nissl staining showed that SZRD could reduce the hippocampal neuron loss in the CA3 and DG subsets of APP/PS1 transgenic mice, so we determined the change of dendritic spines, which are commonly identified using the Golgi-Cox staining. Dendritic spines are one of the essential structures of neurons, which often affect the synaptic function of AD. As shown in Fig. 3, the number of spines and branches were decreased significantly in CA3 and DG subsets in APP/PS1 transgenic mice, in comparison with controls (*P* < 0.01; Fig. 3a–g). However, compared to the APP/PS1 transgenic mice without any treatment, the number of spines and branches in CA3 and DG subsets were markedly increased in the transgenic mice treated with Donepezil and SZRD (*P* < 0.05 or 0.01; Fig. 3a–g). There was no considerable variance in the number of spines and branches between these treatments. Compared to the H-SZRD group, the L-SZRD group had more number of spines and branches in CA3 and DG subsets, but there was no significant difference between these two groups (Fig. 3a–g). Further, to explore the possible mechanism by which SZRD protects synapses, we used Western bolt to detect the protein expression level of synaptic-related proteins, such as synaptophysin (SYN) and postsynaptic density protein 95 (PSD95). We found that SYN and PSD95 in the APP/PS1 transgenic mice’s hippocampus were lowered than the control group. However, the Donepezil and SZRD, especially the L-SZRD, extensively reversed this phenomenon (*P* < 0.05 or 0.01; Fig. 3h–j). These results suggested SZRD could improve the synaptic plasticity of APP/PS1 transgenic mice.

### The SZRD-induced decrease in Aβ plaque deposition in APP/PS1 transgenic mice

**Fig. 4:**
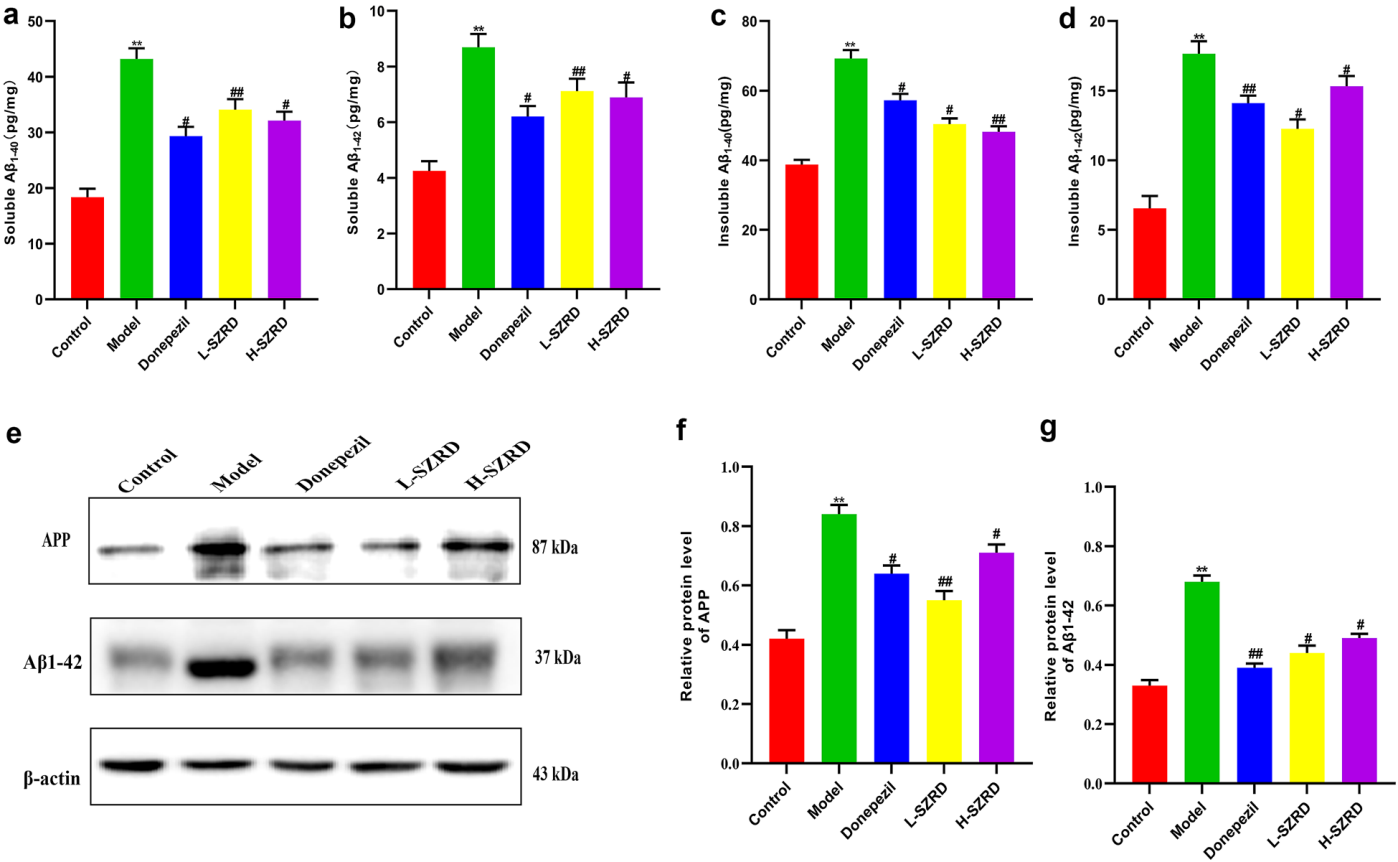
Effect of SZRD on Aβ deposition in the hippocampus of APP/PS1 transgenic mice. **a** The hippocampal content of soluble Aβ_1−40_. **b** The serum content of soluble Aβ_1−42_. **c** The hippocampal content of insoluble Aβ_1−40_. **d** The hippocampal content of insoluble Aβ_1−42_. **e** Examples of original Western blotting bands showing hippocampal expressions of APP and Aβ_1−42_. **f** Relative protein level of APP expression. **g** Relative protein level of Aβ_1−42_ expression. The data are presented as mean ± SEM (n = 6 in each group). **P < 0.01 vs. Control group; ^#^*P* < 0.05 and ^##^*P* < 0.01 vs. Model group

The deposition of Aβ plaque is another essential pathological feature of AD, which is associated with synapse impairment [35]. To investigate the mechanism by which SZRD improves the cognitive dysfunction in APP/PS1 transgenic mice, we examined the protein expressions of Aβ_1−40_ and Aβ_1−42_ in the hippocampus using ELISA. Besides, we also used Western blot to measure the protein expressions of Aβ_1−42_ and APP in the hippocampus. The results of ELISA showed that Donepezil and SZRD, especially the L-SZRD, could ameliorate the protein expressions of soluble Aβ_1−40_ and insoluble Aβ_1−40_ in the hippocampus. These drugs also inhibit the protein expressions of soluble Aβ_1−42_ and insoluble Aβ_1−42_ (*P* < 0.05 or 0.01; Fig. 4a–d). Furthermore, We found that the protein expressions of hippocampal Aβ_1−42_ and APP in APP/PS1 transgenic mice were significantly reduced in Donepezil and SZRD-treated groups, in comparison with the model group without any treatment (*P* < 0.05 or 0.01; Fig. 4e–g). Moreover, more decreases in Aβ_1−42_ and APP were observed in the L-SZRD group than those in the H-SZRD group. These results suggested SZRD could decrease Aβ plaque deposition in the hippocampus of APP/PS1 transgenic mice.

### Effect of SZRD on the Aβ-related α, β, γ secretases and degradation enzyme in the hippocampus of APP/PS1 transgenic mice

**Fig. 5:**
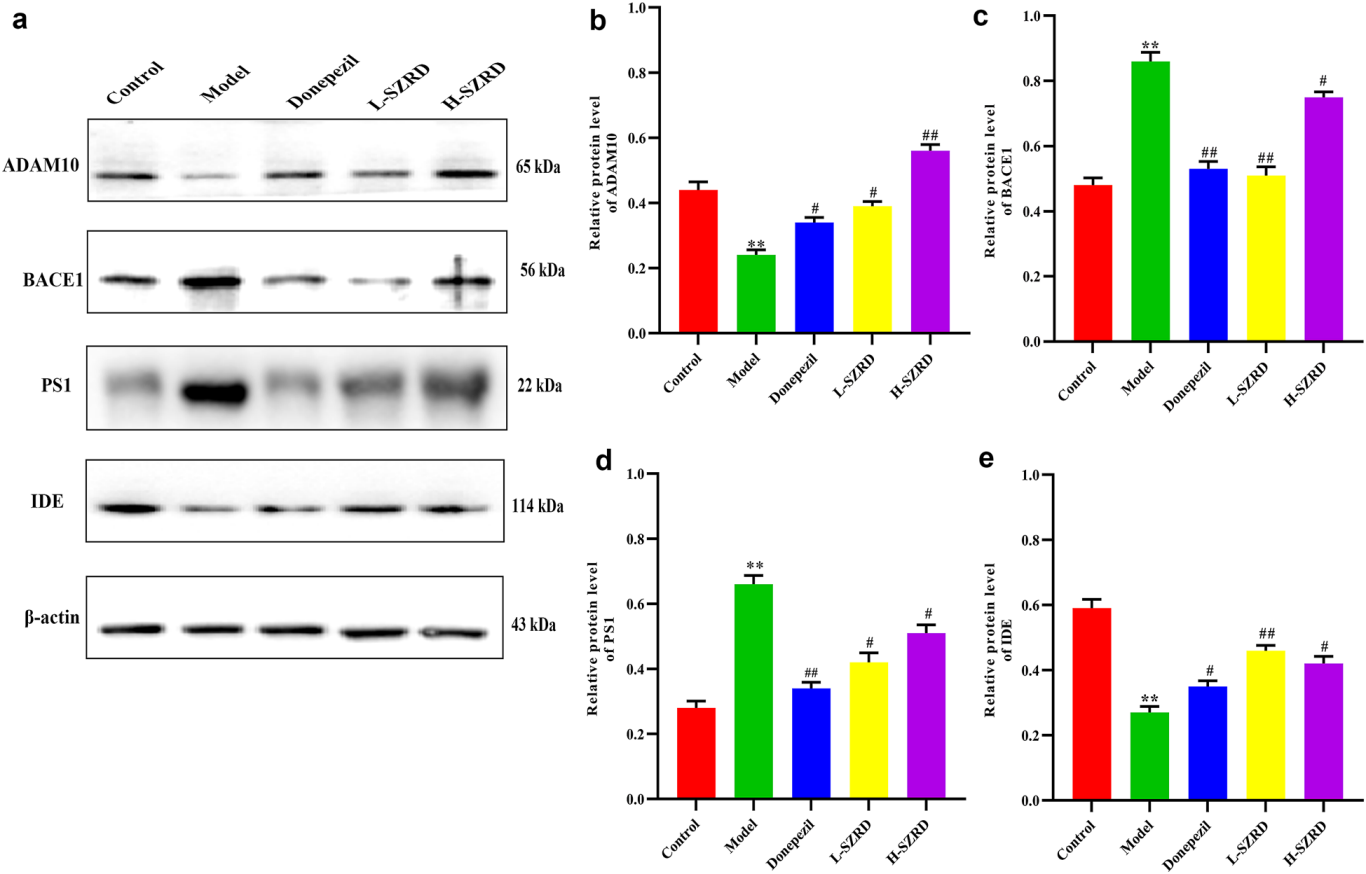
Effect of SZRD on the Aβ-related α, β, γ secretases and degradation enzymein in the hippocampus of APP/PS1 transgenic mice. **a** Examples of original Western blotting bands showing expressions of ADAM10, BACE1, PS1 and IDE in the hippocampus. **b** Relative protein level of ADAM10. **c** Relative protein level of BACE1. **d** Relative protein level of PS1. **e** Relative protein level of IDE. The data are presented as mean ± SEM (n = 6 in each group). **P < 0.01 vs. Control group; ^#^*P* < 0.05 and ^##^*P* < 0.01 vs. Model group

The above experimental results suggested SZRD could decrease Aβ plaque deposition in the hippocampus of APP/PS1 transgenic mice. To further determine whether SZRD can alleviate APP/PS1 transgenic mice’s cognitive deficits by regulating the activities of Aβ secretase and degradation, the expressions of ADAM10, BACE1, PS1 and IDE in the hippocampus were measured with Western blot. We noted that compared to the model group, the levels of BACE1 and PS1 were declined markedly in the hippocampus in the Donepezil and SZRD-treated groups, especially in the L-SZRD group (*P* < 0.05 or 0.01; Fig. 5a, c and d). Also, the expressions of ADAM10 and IDE were notably higher in APP/PS1 mice treated with Donepezil and SZRD than model group (*P* < 0.05 or 0.01; Fig. 5a, b and e). The results showed that SZRD’s inhibition of Aβ deposition may be related to the enhancement of Aβ-related α secretase and degradation enzymes. This effect also may be related to its inhibition of Aβ-related β, γ secretase enzymes.

### Influence of SZRD on inflammation levels in serum and hippocampus of APP/PS1 transgenic mice

**Fig. 6:**
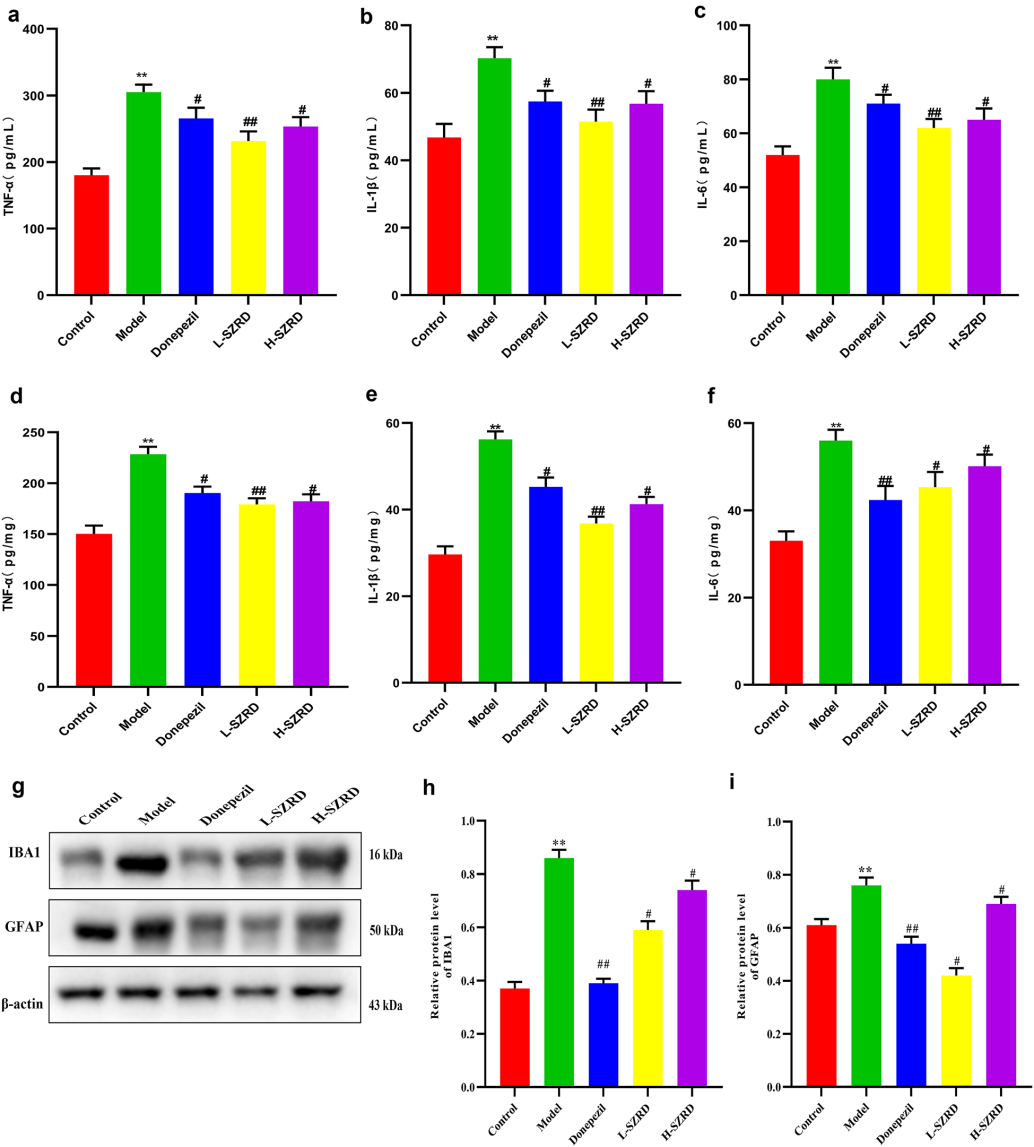
Effect of SZRD on the inflammation levels in serum and hippocampus of APP/PS1 transgenic mice. **a** The serum content of TNF-α. **b** The serum content of IL-1β. **c** The serum content of IL-6. **d** The hippocampal content of TNF-α. **e** The hippocampal content of IL-1β. **f** The hippocampal content of IL-6. **g** Examples of original Western blotting bands showing expressions of IBA1 and GFAP in the hippocampus. **h** Relative protein level of IBA1. **i** Relative protein level of GFAP. The data are presented as mean ± SEM (n = 6 in each group). **P < 0.01 vs. Control group; ^#^*P* < 0.05 and ^##^*P* < 0.01 vs. Model group

The activation of microglia and astrocytes by the Aβ deposition causes extreme release inflammatory factors, including IL-1β, IL-6 and TNF-α in APP/PS1 mice [36]. In this experiment, we measured the serum and hippocampal concentrations of these inflammatory factors using ELISA. Meanwhile, we examined the expressions of the GFAP and IBA1 in the hippocampus using Western blot. We found that the serum and hippocampal levels of IL-1β, IL-6 and TNF-α were much lower in Donepezil and SZRD-treated groups, notably in the L-SZRD group, than those in the model group (*P* < 0.05 or 0.01; Fig. 6a–f). The expressions of hippocampal GFAP and IBA1 in APP/PS1 transgenic mice were significantly enhanced compared to the control group (*P* < 0.05 or 0.01; Fig. 6g–i). After the treatment with SZRD for 4 weeks, the GFAP and IBA1 in the hippocampus of the SZRD-treated groups, especially in the L-SZRD group, were diminished significantly in comparison with the model group without the treatment (*P* < 0.05 or 0.01; Fig. 6g–i). The data demonstrated that the inflammation conditions in the serum and hippocampus were alleviated in APP/PS1 transgenic mice treated with SZRD.

### Down-regulation of the JAK2/STAT3 signaling pathway in the hippocampus by SZRD in APP/PS1 transgenic mice

**Fig. 7:**
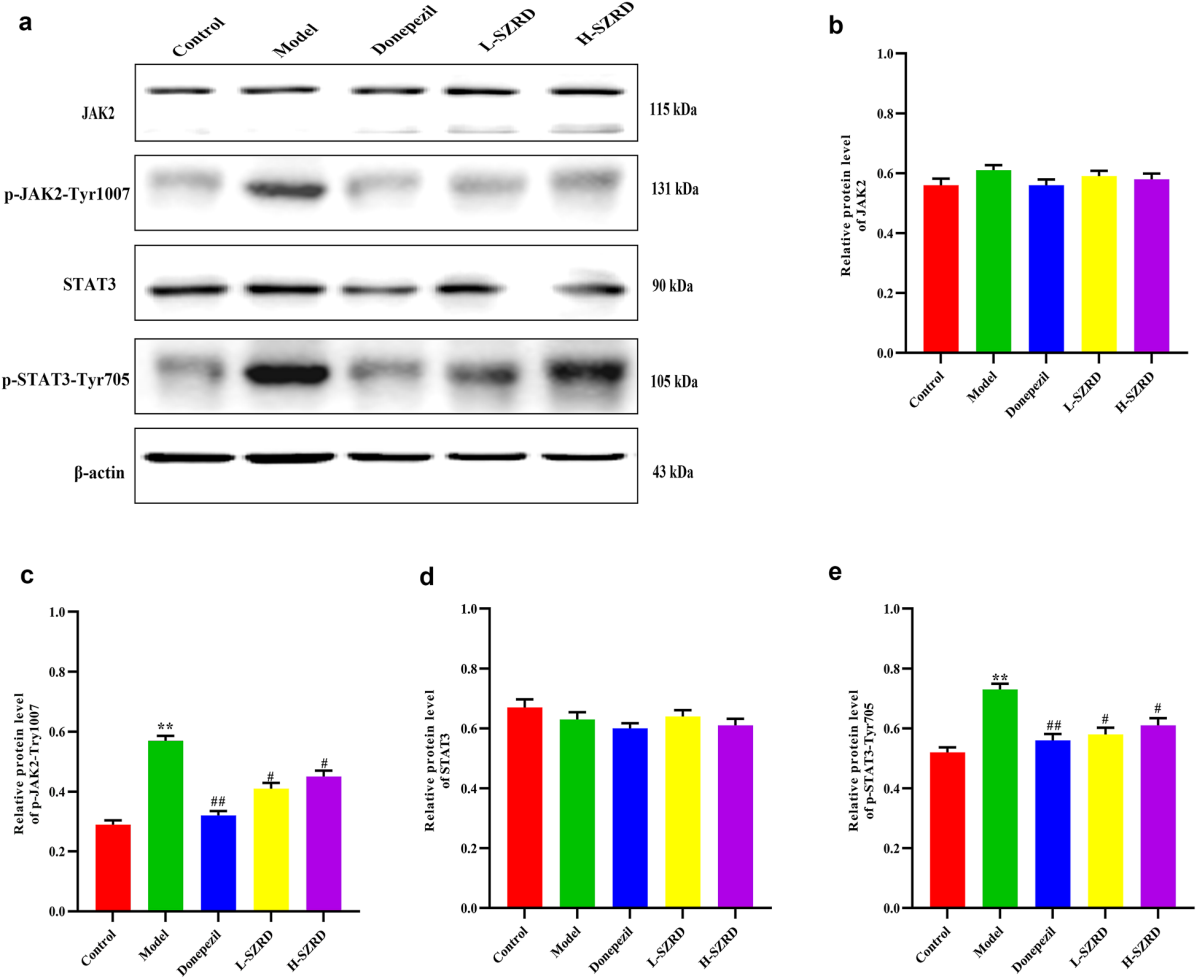
Effect of SZRD on the JAK2/STAT3 signal pathway in the hippocampus of APP/PS1 transgenic mice. **a** Examples of original Western blotting bands showing expressions of JAK2, p-JAK2-Tyr1007, STAT3 and p-STAT3-Tyr705 in the hippocampus. **b** Relative protein level of JAK2. **c** Relative protein level of p-JAK2-Tyr1007. **d** Relative protein level of STAT3. **e** Relative protein level of p-STAT3-Tyr705. The data are expressed as mean ± SEM (n = 6 in each group). **P < 0.01 vs. control group; ^#^*P* < 0.05 and ^##^*P* < 0.01 vs. model group

JAK2/STAT3 signaling pathway, mainly including JAK2/p-JAK2-Tyr1007 and STAT3/p-STAT3-Tyr705, is a crucial inflammatory pathway in the brain [23]. Thus, using Western blot, we examined hippocampal expressions of JAK2, p-JAK2-Tyr1007, STAT3 and p-STAT3-Tyr705 in the mice. We found that the protein levels of p-JAK2-Tyr1007 and p-STAT3-Tyr705 were much higher in APP/PS1 mice than those in the control group (*P* < 0.01; Fig. 7a, c and e). However, after 4 weeks of SZRD treatment, the protein levels of p-JAK2-Tyr1007 and p-STAT3-Tyr705 were reduced markedly in the SZRD-treated groups, particularly in the L-SZRD group, in comparison with the model group (*P* < 0.05 or 0.01; Fig. 6a, c and e). We did not observe among all groups a noticeable difference in the total level of JKA2 and STAT3 of the hippocampus (Fig. 7a, b and d).

## Discussion

Cognitive dysfunction is a typical clinical symptom of AD, and Aβ deposition is an essential pathological feature of AD [37]. The 6-month-old APP/PS1 transgenic mice have apparent perceptive dysfunction and pathological characteristics of Aβ deposition [38]. Thus, this model can mimic AD’s clinical and pathological features and is suitable for AD research. In addition, previous studies have confirmed that APP/PS1 has apparent synaptic dysfunction at age of 6 months, so we used 6-month-old APP/PS1 transgenic mice as AD models in this study [29, 30]. In this study, the learning and memory were assessed in APP/PS1 transgenic mice using the navigation and spatial probe tests. We noticed that, in the navigation test, the escape latency was much longer in APP/PS1 mice than that in the control group. This observation indicated the APP/PS1 mice have visible learning disabilities. In the spatial probe test, compared to the control group, we observed that the times of crossing platform as well as the time staying on the platform were decreased in APP/PS1 transgenic mice compared with the control group, which showed visible memory impairment. However, after treatment with SZRD, we found that SZRD, in particular, the L-SZRD, shortened the escape latency in APP/PS1 transgenic mice. Also, SZRD, especially, the L-SZRD increased the times of crossing platform as well as the time staying on the platform in APP/PS1 transgenic mice. Our findings suggest that SZRD can manage the cognitive dysfunction of mice with AD.

Neurons are crucial to hippocampal-dependent learning and memory [39]. Many studies showed a noticeable loss of hippocampal neurons in patients and experimental animals with AD [40, 41]. Although the mechanisms underlying the loss of neurons in AD are still unclear, there is increasing evidence showing that the neurons in the hippocampus of rats were decreased markedly after Aβ_1−42_ injection into the lateral ventricles [42]. Also, APP/PS1 mice and other AD models have also been confirmed to have a decrease in hippocampal neurons [6, 43]. In this study, we observed a significant decrease in hippocampal neurons of the CA3 and DG subsets in APP/PS1 mice. After treatments with SZRD, the SZRD-treated groups, especially the L-SZRD, increased the neurons in CA3 and DG subsets, which suggested that SZRD may reverse the loss of hippocampal neurons in APP/PS1 transgenic mice.

Synapse serves as the biological basis of cognitive function and participates in hippocampal-dependent learning and memory [44]. Synapse damages, including changes in synaptic structure and function, cause cognitive dysfunction [45]. Many studies have shown that the number of spines and branches in the hippocampus is significantly decreased in the AD models, and cognitive dysfunction can be improved as the number of spines and branches is increased [46–48]. Synaptophysin (SNY) is the biological marker of synaptic formation, and postsynaptic densification protein 95 (PSD95) is one critical protein involved in signal transmission in the synapse [49]. Both SNY and PSD95 play an essential role in synaptic plasticity. With this respect, several studies have demonstrated that the protein expressions of SYN and PSD95 in the AD’s hippocampus were enhanced significantly, and the increase in these protein expressions can restrain cognitive decline [50, 51]. In this study, using Golgi-Cox staining, we found that the number of spines and branches of hippocampal CA3 and DG subsets in APP/PS1 mice was markedly reduced, and the protein levels of SYN and PSD95 in the hippocampus were decreased as well. However, SZRD, particularly L-SZRD increased the number of spines and branches of CA3 and DG subsets as well as protein expressions of SYN and PSD95 in the hippocampus. Our findings suggest that SZRD may improve synaptic plasticity in APP/PS1 mice.

Aβ deposition is one of the typical pathological features of AD, of which Aβ_1−40_ and Aβ_1−40_ are the core components [35]. In this study, we used ELISA to measure the protein expressions of Aβ_1−40_ and Aβ_1−42_ in the hippocampus. Our research showed that SZRD could reduce the expression levels of both soluble and insoluble Aβ_1−40_ and Aβ_1−42_ in the hippocampus of mice. In this study, we also found that the protein expressions of Aβ_1−42_ and APP in the hippocampus were significantly enhanced in APP/PS1 mice, contrasted to controls, indicating hippocampal Aβ deposition in the mice with AD. After treatments with SZRD, notably, L-SZRD, the hippocampal expressions of Aβ_1−42_ and APP were decreased obviously in APP/PS1 mice, which indicated that SZRD might inhibit Aβ generation. Aβ deposition depends on the hydrolysis of APP, which is regulated through α, β, γ secretase [52]. The α-secretase is mainly composed of the ADAM family [42]. ADAM10 can reduce the pathological damage of AD by inhibiting the production of Aβ_1−42_. BACE1 and PS1 are parts of β and γ secretase, respectively. They can promote the production of Aβ_1−42_. Therefore, inhibiting the expression of BACE1 and PS1 can reduce Aβ production. IDE has the function to degrade Aβ, which can clear the Aβ deposition in AD. Meanwhile, we observe that the protein expression levels of BACE1 and PS1 were augmented significantly in APP/PS1 mice, while the protein levels of ADAM10 and IDE were markedly reduced. These changes in APP/PS1 mice were reversed obviously following the treatment with SZRD. As such, our results suggest that SZRD can reduce Aβ deposition by enhancing α-secretase and degrading enzyme and reducing β-secretase and γ-secretase.

It remains unclear how inflammatory responses cause brain injury. Some investigators have demonstrated that inflammation contributes to AD’s pathogenesis [53–55]. The inflammation in AD is associated with multiple factors. Aβ deposition is considered an essential cause. In this respect, Aβ deposition induces microglia’s activation to generate pro-inflammatory factors, such as IL-1β, IL-6 and TNF-α, which finally cause neuroinflammation [56]. In the present study, we found that the serum and hippocampal concentrations of IL-1β, IL-6 and TNF-α were much upper in the APP/PS1 mice than those in controls. Besides, the protein expressions of hippocampal IBA1 and GFAP were increased substantially in APP/PS1 mice. These results indicate neuroinflammation occurs in mice with AD. However, after the APP/PS1 mice were treated with SZRD, we observed a significant reduction in IL-1β, IL-6 and TNF-α in serum and hippocampus, as well as the protein expressions of IBA1 and GFAP in the hippocampus. These findings suggest that SZRD may alleviate neuroinflammation in the serum and hippocampus of AD mode.

The signaling pathway associated with JAK2/STAT3 is closely related to inflammation, oxidative stress and apoptosis, which mainly affects the AD’s pathological process through inflammation regulation [23]. JAK2 represents the Janus kinase (JAK) family and is activated at the Tyr1007 site by IL-1β, IL-6 and TNF-α. Phosphorylated JAK2 promotes the activation of STAT3 at the Tyr705 site, which finally leads to neuroinflammation and apoptosis [57–59]. There is increasing evidence showing that the JAK2/STAT3 signaling pathway is activated in AD, and the inhibition of this signaling pathway can alleviate neuroinflammation in AD [25, 60]. In this study, we observed that in comparison with the control group, the expressions of hippocampal p-JAK2-Tyr1007 and p-Tyr705-STAT3 were augmented extensively in APP/PS1 mice. At the same time, the expressions of JAK2 and STAT3 were unchanged. These results demonstrated the JAK2/STAT3-associated signaling pathway in the hippocampus is initiated in mice with AD. After the treatment with SZRD, the protein expression of p-JAK2-Tyr1007 and p-Tyr705-STAT3 in the hippocampus of the SZRD-treated group was significantly decreased. Our findings suggest that SZRD may down-regulate the JAK2/STAT3 signaling pathway in the hippocampus to alleviate neuroinflammation in AD.

## Conclusions

In summary, our study shows that SZRD can alleviate cognitive impairment by reducing neuronal loss and synaptic damage in APP/PS1 transgenic mice. Also, SZRD can attenuate neuroinflammation and inhibit the excitation of microglia in APP/PS1 mice, which likely is associated with its blockade of the JAK2/STAT3-related signaling pathway. SZRD’s anti-inflammatory action suggests that SZRD may have a beneficial effect on AD.

## Data Availability

The data used to support the current study are available from the corresponding author on reasonable request.

## Abbreviations

SZRD: Suan-Zao-Ren Decoction
WB: Western blot
ELISA: Enzyme-linked immunosorbent assay
IL-6: Interleukin-6
IL-1β: Interleukin-1β
TNF-α: Tumor necrosis factor-α
Aβ_1−40_: Amyloid peptide_1−40_
Aβ_1−42_: Amyloid peptide_1−42_
APP: Amyloid precursor protein
ADAM10: A disintegrin and metalloproteinase 10
BACE1: β-secretase 1
PS1: Presenilin 1
IDE: Insulin-degrading enzyme
IBA1: Ionized calcium binding adaptor molecule 1
GFAP: Glial fibrillary acidic protein
PSD95: Postsynaptic density proteins 95
SYN: Synaptophysin
JAK2: Janus kinase 2
STAT3: Signal transducer and activator of transcription 3
